# Unraveling the Skeletal Growth-Promoting Mechanism of the Seahorse *Hippocampus erectus*: From Active Fraction Screening to Signaling Pathway Regulation

**DOI:** 10.3390/cimb48070678

**Published:** 2026-06-30

**Authors:** Lianghua Huang, Zhaoji Pan, Meng Bai, Jiyan Guo, Jian Xiao, Chenghai Gao

**Affiliations:** 1 Institute of Marine Drugs, Guangxi University of Chinese Medicine, Nanning 530200, China; huanglh@gxtcmu.edu.cn (L.H.); pzj199910@163.com (Z.P.); xxbai2014@163.com (M.B.); gjyfjq2026@163.com (J.G.); 2 Guangxi Key Laboratory of Marine Drugs, Guangxi University of Chinese Medicine, Nanning 530200, China; 3 University Engineering Research Center of High-Efficient Utilization of Marine Traditional Chinese Medicine Resources, Nanning 530200, China; 4 Engineering Research Center of Innovative Drug of Traditional Chinese and Zhuang Yao Medicine, Ministry of Education, Guangxi University of Chinese Medicine, Nanning 530200, China; 5 Guangxi Engineering Research Center for High-Value Utilization of Guangxi-Produced Authentic Medicinal Herbs, Nanning 530200, China; 6 Characteristic Chinese Traditional & Ethnic Medicine Engineering Research Center for Guangxi Universities, Nanning 530200, China

**Keywords:** *Hippocampus erectus*, aqueous extract fractions, osteogenic activity, molecular mechanism

## Abstract

As a traditional element of Chinese medicine, *Hippocampus erectus* is well known for promoting adolescent growth, yet its active fractions and underlying molecular mechanisms remain unclear. In this study, the aqueous extract of *H. erectus* was subjected to in vitro simulated gastrointestinal digestion and ultrafiltration to separate three molecular weight fractions (<10 kDa, 10–30 kDa, >30 kDa). Their chemical profiles were characterized, and osteogenic activities were systematically evaluated using cell assays, a juvenile rat model, and integrated transcriptomics and data-independent acquisition (DIA) proteomics. Results revealed that chemical profiling showed the >30 kDa fraction was mainly composed of hemocyanin subunits, and the 10–30 kDa fraction was enriched in growth-related amino acids and steroid derivatives; functionally, the 10–30 kDa fraction promoted preosteoblast proliferation and early differentiation via enhanced alkaline phosphatase (ALP) activity, while the >30 kDa fraction dominated late osteoblast maturation and mineralization. Both fractions significantly increased rat body and bone length by expanding growth plate proliferative zones and elevating serum insulin-like growth factor-1 (IGF-1)/bone morphogenetic protein-2 (BMP-2) levels. Transcriptomic and proteomic analyses identified vascular endothelial growth factor (VEGF), Wingless-related integration site (Wnt), phosphatidylinositol 3-kinase-protein kinase B (PI3K-Akt), and extracellular matrix (ECM)–receptor interaction as potential core regulatory pathways. Integrated multi-omics analysis further confirmed Frizzled-related protein B (*Frzb*) and AKT1 substrate 1 (*Akt1s1*) as candidate key regulatory targets enriched in the Wnt and adenosine monophosphate-activated protein kinase (AMPK) signaling pathways. These findings elucidate the multi-fraction, multi-pathway mechanism of *H. erectus* in promoting skeletal development, providing scientific evidence for its traditional use and a theoretical basis for growth-promoting functional food development.

## 1. Introduction

*Hippocampus erectus*, a traditional Chinese marine medicine, has been historically employed to “warm the kidney and invigorate yang” and to “dissipate stagnation and reduce swelling.” It was first documented in Ben Cao Shi Yi (Supplement to the Materia Medica) [1]. In the coastal areas of China, it is a common folk practice to include *H. erectus* in culinary preparations, such as “Seahorse Chicken Soup”, to support growth and development in adolescents through dietary intervention. However, despite its long-standing and widespread traditional use, the pharmacodynamic basis and precise molecular mechanisms responsible for its growth-promoting effects remain unclear. Therefore, a systematic scientific investigation is needed to elucidate these aspects, as the current knowledge gap hinders the standardized development and application of *H. erectus* in functional products aimed at adolescent growth and in the broader health industry.

In recent years, the growing interest in bioactive compounds from marine organisms has drawn particular attention to seahorse medicinal materials, which are rich in proteins, peptides, amino acids, and various trace elements. Among these fractions, proteins and polypeptides, known for their high bioavailability and potent biological activities, are considered primary bioactive constituents [2,3]. Marine-derived peptides exhibit great potential in regulating osteoblast behavior and mimicking the functions of bone matrix collagen due to their unique amino acid composition, especially their high content of proline and hydroxyproline [4]. In addition, marine-derived bioactive peptides not only possess superior tissue penetration capacity, but also precisely promote the osteogenic differentiation of mesenchymal stem cells and inhibit osteoclast activity by mimicking or modulating key osteogenic signaling pathways [5].

During simulated gastrointestinal digestion, macromolecular proteins are hydrolyzed into peptide fragments with different molecular weights. The biological activities of these peptides are closely linked to their molecular weights, amino acid sequences, and spatial conformation [6,7,8]. Consequently, peptides with different molecular weights may exhibit markedly distinct biological functions [6,8]. For instance, low-molecular-weight peptides (1–3 kDa) derived from marine organisms, such as those extracted from *Nibea japonica* [9], *Tympanotonus fuscatus* var. radula (Linnaeus), and *Pachymelania aurita* (Müller) [10], often exhibit antioxidant and anti-inflammatory properties. In contrast, medium- and high-molecular-weight peptide fragments (>10 kDa) are frequently implicated in the regulation of complex physiological processes, including cell proliferation and differentiation [11,12].

Linear bone growth is a complex, multistage biological process primarily driven by the proliferation and differentiation of growth plate chondrocytes, followed by osteogenic mineralization. This process is tightly regulated by various growth factors and their associated signaling pathways [13,14]. Insulin-like growth factor-1 (IGF-1) plays a key role in promoting chondrocyte proliferation and enhancing osteoblast activity. Bone morphogenetic protein-2 (BMP-2) facilitates the differentiation of mesenchymal stem cells into osteoblasts. Core signaling pathways, such as vascular endothelial growth factor (VEGF), phosphatidylinositol 3-kinase-protein kinase B (PI3K-Akt), and Wingless-related integration site (Wnt), form an essential regulatory network that governs chondrocyte fate and skeletal development [15,16]. Previous studies have indicated that bioactive peptides derived from marine organisms can modulate bone growth by targeting growth factors and signaling pathways. For instance, sea cucumber peptide (SJP) has been shown to promote proliferation, alkaline phosphatase (ALP) activity, and mineralization in MC3T3-E1 cells and stimulate osteoblast differentiation via Sma and Mad homologs (Smad) pathway activation [17]. Similarly, *Mytilus edulis* protein hydrolysate (BMPH) enhances osteoblast differentiation in mouse mesenchymal stem cells by upregulating bone morphogenetic protein expression [18]. However, the pharmacodynamic basis and molecular mechanisms underlying the growth-promoting activity of *H. erectus* remain unknown.

In view of the above, the current investigation used dried bodies of *H. erectus* as the starting material. After aqueous extraction and in vitro simulated gastrointestinal digestion, ultrafiltration was applied to separate and yield three molecular weight fractions: <10 kDa, 10–30 kDa, and >30 kDa. Using the 3-(4,5-dimethylthiazol-2-yl)-2,5-diphenyltetrazolium bromide (MTT) assay, ALP staining, and Alizarin Red S staining, we systematically assessed the influence of each fraction on the proliferation, differentiation, and mineralization of MC3T3-E1 preosteoblasts. Additionally, a juvenile rat growth model was established to examine the regulatory effects of bioactive fractions on longitudinal bone growth and serum levels of growth-related factors. Finally, transcriptomics, proteomics, and combined analyses were performed to identify the key signaling pathways involved in the osteogenic and growth-promoting activities of the active fractions. This study provided scientific evidence for the traditional use of *H. erectus* in “promoting growth” and established a theoretical basis for its development as a growth-promoting product for adolescents and its extensive application in the health industry.

## 2. Materials and Methods

### 2.1. Preparation of H. erectus Aqueous Extract (HAE)

Dried *H. erectus* specimens of uniform size (body length: 12 ± 1 cm; official batch No. BS-HM-003, Guangxi Baishen Biotechnology Co., Ltd., Beihai, Guangxi, China) were cut into small pieces (approximately 0.5 cm^3^) and ground using an FZ102 universal pulverizer (Tianjin Taisite Instrument Co., Ltd., Tianjin, China). The resulting powder was passed through a No. 2 sieve (aperture: 850 μm) for size separation purposes. Deionized water was added at a solid-to-liquid ratio of 1:15 (g/mL), and the mixture was extracted in an 80 °C water bath for 2 h. The extract was filtered through a 200-mesh nylon sieve (aperture: 75 μm), and the residue was subjected to three additional extractions under identical conditions. The combined filtrates were concentrated under reduced pressure at 60 °C to a relative density of 1.25 g/mL using an RE-52AA rotary evaporator (Shanghai Yarong Biochemical Instrument Co., Ltd., Shanghai, China). The concentrated extract was then freeze-dried at −50 °C for 24 h using an FD-1A-50 freeze-dryer (Beijing Boyikang Experimental Instrument Co., Ltd., Beijing, China). The final powder was sealed and stored at −20 °C until further analyses.

### 2.2. Simulated Gastric and Intestinal Digestion

An in vitro simulated gastrointestinal digestion model was established as described by Gong et al. [19] with minor modifications. Artificial gastric juice was prepared by dissolving 2.0 g of sodium chloride, 3.2 g of pepsin (1:10,000, Cat. No. 24240907006, Solarbio Science & Technology Co., Ltd., Beijing, China), and 7.0 mL of concentrated hydrochloric acid (Cat. No. 2022060101, Guangdong Guanghua Sci-Tech Co., Ltd., Shantou, Guangdong, China) in deionized water, which was then diluted to a final volume of 1 L and adjusted to pH 2.0. Artificial intestinal juice was prepared by dissolving 6.8 g of potassium dihydrogen phosphate in 250 mL of ultrapure water, followed by the addition of 77 mL of 0.2 M sodium hydroxide solution (Cat. No. 20241018, Guangdong Guanghua Sci-Tech Co., Ltd., Shantou, Guangdong, China) and 10.0 g of trypsin (1:250, Cat. No. 24240807011, Solarbio Science & Technology Co., Ltd., Beijing, China). The mixture was thoroughly mixed, diluted to 1 L, and the pH was adjusted to 6.8. A precisely weighed quantity of freeze-dried aqueous extract of *H. erectus* was dissolved in 500 mL of artificial gastric juice and incubated at 37 °C with shaking at 200 rpm for 3 h in a thermostatic incubator (Shanghai Zhicheng Analytical Instrument Manufacturing Co., Ltd., Shanghai, China). The enzyme activity was terminated by heating the mixture in a boiling water bath at 100 °C for 10 min.

After the mixture cooled to room temperature, its pH was adjusted to 6.8 ± 0.1 with 1 M NaOH. The mixture was then centrifuged at 8000 rpm and 4 °C for 10 min, and the resulting supernatant was collected and stored at 4 °C until further analyses. For intestinal digestion, the gastric digestion supernatant was combined with artificial intestinal juice in a 1:1 volume ratio and incubated under identical shaking conditions (37 °C, 200 rpm) for an additional 4 h. Enzyme inactivation and centrifugation were performed as previously described. The final supernatant was collected for ultrafiltration, bioactivity assessment, and other analyses.

### 2.3. Sodium Dodecyl Sulfate-Polyacrylamide Gel Electrophoresis (SDS-PAGE) Analysis

SDS-PAGE was performed as described by Tian et al. [20]. A 4.5% stacking gel and 10% resolving gel were prepared as described previously. For sample preparation, 20 mg of freeze-dried powder was dissolved in loading buffer and equilibrated at 4 °C for 24 h to ensure complete dissolution. Subsequently, 10 μL of each sample solution was loaded into the gel wells along with 5 μL of the protein marker. Electrophoresis was initially performed at a constant voltage of 70 V for 30 min to allow migration through the stacking gel. Once the bromophenol blue tracking dye reached the stacking-resolving gel interface, the voltage was increased to 110 V and maintained for approximately 60 min. Electrophoresis was stopped when the dye front was approximately 10 mm from the bottom of the gel. Following separation, the gels were stained with Coomassie Brilliant Blue R-250 for 4 h. Destaining was performed using a methanol/acetic acid/water solution (4:1:5, *v*/*v*/*v*), with the destaining solution refreshed every 2 h until a clear background was obtained.

### 2.4. High Performance Liquid Chromatography (HPLC) Analysis of the Four Fractions

All sample solutions were filtered through a 0.45 µm membrane filter prior to analysis. The four fractions were analyzed on a Shimadzu LC-2030C 3D Plus HPLC system (Shimadzu Corporation, Kyoto, Japan) equipped with a PDA detector and a BioPulite XP Protein tC18 column (Shimadzu Corporation, Kyoto, Japan; 250 × 4.6 mm, 5 μm, 300 Å). The analysis was performed at 30 °C with a flow rate of 1.0 mL/min. The mobile phase consisted of 0.1% acetic acid in water (A) and acetonitrile (B), using the following linear gradient elution: 0–10 min, 5% B; 10–15 min, 5–55% B; 15–25 min, 55% B; 25–30 min, 55–100% B; 30–40 min, 100% B; 40–45 min, 100–5%; 45–55 min, 5% B. The injection volume was 10 µL, and UV detection was carried out at 254 nm.

### 2.5. Ultra-High Performance Liquid Chromatography-Quadrupole-Exactive Orbitrap Mass Spectrometry (UHPLC-Q-Exactive Orbitrap MS) Analysis

UHPLC analysis was performed using a Vanquish UPLC system (Thermo Fisher Scientific, Waltham, MA, USA) equipped with an online vacuum degasser, a quaternary pump, and an automatic sampler. Chromatographic separation was achieved on a Waters HSS T3 column (Waters, Milford, MA, USA; 100 × 2.1 mm, 1.8 μm). The mobile phase consisted of ultra-pure water containing 0.1% formic acid (phase A) and acetonitrile containing 0.1% formic acid (phase B). The elution was performed at a constant flow rate of 0.3 mL/min with a column temperature of 40 °C, and the injection volume was 2 μL. The gradient elution program was as follows: 0–1 min, 100% A (0% B); 1–4 min, 100–40% A (0–60% B); 4–6.5 min, 40–5% A (60–95% B); 6.5–6.6 min, 5–100% A (95–0% B); 6.6–8 min, 100% A (0% B). Mass spectrometry detection was conducted in both positive and negative ionization modes using a heated electrospray ionization source with parameters set as sheath gas 40 arb, auxiliary gas 10 arb, spray voltage +3000 V/−2800 V, ion source temperature 350 °C, ion transfer tube temperature 320 °C, and data were acquired in full-scan data-dependent tandem mass spectrometry (Full-ms-ddMS2) mode with a primary scan range of 70–1050 *m*/*z* and resolutions of 70,000 for full MS and 17,500 for MS/MS. Raw data were collected by Xcalibur 4.1, processed with Progenesis QI, and metabolites were identified against commercial and SanShu Bio self-built animal metabolite standard libraries following the Metabolomics Standards Initiative confidence level criteria.

### 2.6. Ultrafiltration Separation and Fraction Preparation

Ultrafiltration and fraction separation were performed as described by Jiang [12] with minor modifications. The simulated gastrointestinal digest was initially pre-filtered using a 0.45 μm polyvinylidene fluoride (PVDF) membrane (Merck Millipore, Billerica, MA, USA). Subsequently, sequential ultrafiltration was performed using an Amicon Ultra-15 device (Merck Millipore, Billerica, MA, USA) equipped with molecular weight cut-off (MWCO) membranes of 30 and 10 kDa. The process was conducted under a nitrogen pressure of 0.2 MPa at 4 °C and halted when the retentate volume decreased to approximately 25% of the initial volume, at which point the filtrate was collected. The procedure was repeated twice. Three molecular weight fractions were obtained: high (>30 kDa), medium (10–30 kDa), and low (<10 kDa). Each fraction was then freeze-dried at −50 °C for 48 h in an FD-1A-50 freeze dryer (Beijing Boyikang Experimental Instrument Co., Ltd., Beijing, China). The resulting powders were hermetically sealed and stored at −80 °C in an ultralow-temperature freezer (Qingdao Haier Biomedical Co., Ltd., Qingdao, Shandong, China) until further analyses.

### 2.7. Evaluation of Osteogenic Activity In Vitro

MC3T3-E1 preosteoblasts (Cat. No. CL-0378, Procell Life Science & Technology Co., Ltd., Wuhan, Hubei, China) were maintained in α-minimum essential medium (Cat. No. 6124465, Thermo Fisher Scientific, Waltham, MA, USA) containing 10% fetal bovine serum (FBS, Cat. No. SA240411, Procell Life Science & Technology Co., Ltd., Wuhan, Hubei, China) and 1% penicillin-streptomycin (100×, Cat. No. 240012016, Solarbio Science & Technology Co., Ltd., Beijing, China) at 37 °C in a humidified atmosphere of 5% CO_2_. Cells between passages 3 and 10 were used in all experimental procedures. Osteogenic differentiation was induced using complete α-MEM supplemented with 10 nM dexamethasone, 50 μg/mL L-ascorbic acid-2-phosphate, and 10 mM β-glycerophosphate (components from osteogenic differentiation induction kit, Cat. No. WH4024D275, Procell Life Science & Technology Co., Ltd., Wuhan, Hubei, China).

Cell proliferation was evaluated using the MTT colorimetric assay (Cat. No. 240009006, Solarbio Science & Technology Co., Ltd., Beijing, China). MC3T3-E1 cells were plated in 96-well plates at a density of 8 × 10^3^ cells per well and pre-cultured for 12 h to facilitate attachment. The cells were then exposed to different concentrations (25–200 μg/mL) of each molecular weight fraction for 48 h. After incubation, 20 μL of MTT solution (5 mg/mL) was added to each well and incubated for 3 h in the dark. The medium was carefully aspirated, and 150 μL of dimethyl sulfoxide (DMSO) was added to each well to solubilize the formazan crystals. Absorbance was measured at 570 nm using a microplate reader (PerkinElmer, Inc., Waltham, MA, USA). All experiments were conducted in six replicates per condition (*n* = 6).

ALP activity was assessed in MC3T3-E1 cells seeded at 1 × 10^5^ cells per well in 24-well plates. After 24 h of cell attachment, the culture medium was replaced with osteogenic induction medium containing 50 or 200 μg/mL of each test fraction. The induction medium was changed every 48 h. 5-bromo-4-chloro-3-indolyl phosphate/nitro blue tetrazolium (BCIP/NBT) staining (Cat. No. A011241212, Beyotime Biotechnology Co., Ltd., Shanghai, China) was performed on days 3 and 7 of induction. The integrated optical density (IOD) of the stained areas was measured using ImageJ software (version 1.53). All experiments were performed in triplicate, with six random fields analyzed for each sample (*n* = 3).

To detect mineralized nodules, cells were seeded in 6-well plates at a density of 2.0 × 10^5^ cells per well. After 48 h, when approximately 80% confluence was reached, the following treatments were applied: the blank group received complete culture medium, the model group was treated with osteogenic induction medium, and the treatment groups were exposed to osteogenic induction medium supplemented with 200 μg/mL of each fraction. The medium was refreshed every 48 h. After 21 days of induction, mineralized nodule formation was evaluated using Alizarin Red S staining (Alizarin Red S method, Cat. No. C110485S, Beyotime Biotechnology Co., Ltd., Shanghai, China). Images were acquired using a light microscope (Leica Microsystems, Beijing, China) at 10× magnification, and the percentage of mineralized nodule-positive area (Positive Area %) was quantified using ImageJ software (version 1.53).

### 2.8. Animal Experiment Design

A total of 60 specific pathogen-free (SPF) 4-week-old male Wistar rats (body weight 80 ± 10 g; Beijing Speifei Biotechnology Co., Ltd., Beijing, China) were acclimatized for 5 days under controlled environmental conditions (temperature 23 ± 1 °C; relative humidity 55 ± 5%) with ad libitum access to standard diet and water. Subsequently, the rats were randomly divided into five groups (*n* = 12 per group): (1) Blank control group: normal saline was administered via oral gavage (1 mL/100 g body weight, BW). (2) Positive control group: subcutaneously administered recombinant human growth hormone (rhGH, Cat. No. 202404K023, Shanghai United Cell Biotechnology Co., Ltd., Shanghai, China) at a dose of 200 μg/kg BW per day. (3) Low-molecular-weight fraction group (<10 kDa), (4) medium-molecular-weight fraction group (10–30 kDa), and (5) high-molecular-weight fraction group (>30 kDa): each group was intragastrically administered the corresponding ultrafiltration fraction derived from in vitro simulated gastrointestinal digests of *H. erectus* aqueous extract at a dose of 1000 mg/kg BW per day for 21 consecutive days. Daily food intake was recorded throughout the study, and body weight was measured at regular intervals. Snout-to-anus length (SNL) and tail length (TL, measured from the anus to the tail tip) were assessed every 72 h using a digital caliper (precision 0.01 mm, Mitutoyo Corporation, Kawasaki, Japan).

### 2.9. Sample Collection and Analysis

At the end of the treatment period, the rats were fasted for 12 h with free access to water and then sacrificed. Anesthesia was induced by intraperitoneal injection of a 10% chloral hydrate solution (0.4 mL/100 g body weight). Blood samples (approximately 5 mL) were collected from the abdominal aorta and placed in vacuum tubes without anticoagulants. After being kept at room temperature for 1–2 h, the samples were centrifuged at 3000 rpm and 4 °C for 10 min to obtain serum. The serum was aliquoted and stored at −80 °C until further analysis. Serum concentrations of IGF-1, BMP-2, ALP, and insulin-like growth factor binding protein 3 (IGFBP-3) (Cat. No. 20241110, Wuhan Boster Biological Technology Co., Ltd., Wuhan, Hubei, China) were measured using commercial enzyme-linked immunosorbent assay (ELISA) kits according to the manufacturer’s protocols [21,22]. For histological evaluation, immunohistochemical (IHC) staining was performed to assess the expression of IGF-1 (Abcam plc, Cambridge, UK, Cat. No. ab9572) and osteocalcin (OCN; Abcam plc, Cambridge, UK, Cat. No. ab93876) in growth plate tissues. Semi-quantitative analysis was performed using the H-score method with QuPath software (version 0.3.2) [23,24].

Following blood collection, the bilateral femurs and tibias were promptly dissected, and the adherent muscles were removed from the bones. The right femur and tibia were dried at 105 °C until a stable weight was reached. The dry weight was documented, and the length was measured using a vernier caliper (with an accuracy of 0.01 mm). The proximal epiphysis of the right tibia was fixed with 4% paraformaldehyde (PFA, Sigma-Aldrich, St. Louis, MO, USA, Cat. No. P6148) for 48 h, decalcified with ethylenediaminetetraacetic acid (EDTA, Sigma-Aldrich, Cat. No. E9884, pH 7.4) for 21 d, dehydrated with gradient ethanol, and embedded in paraffin blocks. Sagittal section slices with a thickness of 4 μm were prepared. The morphology of the growth plate was observed under a microscope after hematoxylin and eosin (HE) staining. In accordance with the method proposed by Lee et al. [25], six measurement points were selected in the middle one-third area of the inner and outer sides of the growth plate using ImageJ, and the mean value was considered the total height of the growth plate. The heights of the proliferative and hypertrophic zones were measured separately (*n* = 6).

### 2.10. Transcriptomic Analysis

The right femurs were collected from rats in the blank control group, 10–30 kDa group, and >30 kDa group (*n* = 4 per group), immediately snap-frozen in liquid nitrogen, and stored at −80 °C in an ultra-low temperature freezer. All samples were submitted to Guangzhou Genedenovo Biotechnology Co., Ltd., Guangzhou, Guangdong, China for transcriptomic analyses. Total RNA was isolated using TRIzol reagent. RNA purity (A260/A280 > 1.9) was evaluated by 1% agarose gel electrophoresis and verified using a NanoPhotometer NP80 spectrophotometer (Implen, Munich, Germany). RNA concentration was measured using a Qubit 2.0 Fluorometer, with an integrity threshold set at RNA integrity number (RIN) > 8.0, and RNA integrity was assessed using an Agilent 2100 Bioanalyzer. Complementary DNA (cDNA) libraries were constructed using NEBNext Ultra II RNA Library Prep Kit for Illumina. Paired-end sequencing (150 bp read length) was performed using an Illumina NovaSeq 6000 platform (Illumina, Inc., San Diego, CA, USA). Raw sequencing reads underwent quality control using Trimmomatic to remove adapter sequences and low-quality reads.

Clean reads were mapped to the rat reference genome (Rat Genome Assembly Rnor 6.0) using HISAT2. Gene expression levels were quantified, and differentially expressed genes (DEGs) were identified using the DESeq2 R package, applying thresholds of |log_2_ fold change (log_2_FC)| ≥ 0.585 and false discovery rate (FDR) < 0.05. Gene Ontology (GO) and Kyoto Encyclopedia of Genes and Genomes (KEGG) pathway enrichment analyses were performed using R software (version 4.3.1) with the clusterProfiler R package (v4.0), with an FDR cutoff of ≤0.05. To validate the transcriptomic results, quantitative real-time polymerase chain reaction (qRT-PCR) was used to measure the relative expression levels of four key bone growth-related genes: *IGF-1*, *BMP-2*, liver/bone/kidney (*ALPL*), and *IGFBP-3*. Gene-specific primers were custom-designed and synthesized, and qRT-PCR assays were conducted on a real-time fluorescence quantitative PCR system by Guangzhou Genedenovo Biotechnology Co., Ltd., Guangzhou, Guangdong, China. The primer sequences are listed in Table 1.

### 2.11. Proteomics Analysis

The right femurs of rats in the blank group and the 10–30 kDa group (*n* = 4) were collected, frozen in liquid nitrogen, and stored at −80 °C. Subsequently, these samples were sent to Guangzhou Genedenovo Biotechnology Co., Ltd. for data-independent acquisition (DIA) proteomic sequencing. For sample pretreatment, an iST kit (96×, PreOmics GmbH, Martinsried, Germany) was used for protein denaturation, reduction, alkylation, trypsin digestion, and peptide desalting. The specific procedures were as follows: An appropriate quantity of protein was added to the lysis buffer. The mixture was heated at 95 °C at a rotational speed of 1000 rpm for 10 min. After cooling, trypsin digestion buffer was added, and the mixture was incubated at 37 °C with a rotational speed of 500 rpm for 2 h. After the reaction was terminated, desalting was performed using an iST cartridge. The peptide segments were eluted, vacuum-dried, and stored at −80 °C. The desalted and freeze-dried peptide segments were re-solubilized in a 0.1% formic acid aqueous solution. Analysis was performed using a liquid chromatography-tandem mass spectrometry (LC-MS/MS) system consisting of an UltiMate 3000 and timsTOF Pro2 mass spectrometer (Bruker Daltonics, Bremen, Germany). The loading quantity was 200 ng/well. The gradient separation lasted for 60 min (starting with 4% of the B phase, increasing to 28% in 25 min, then to 44% in 10 min, to 90% in 10 min and maintaining for 7 min, and finally returning to 4% for 8 min). The column temperature was set to 50 °C, and the flow rate was 400 nL/min. The mass spectrometry collected DIA data in the data-independent acquisition parallel accumulation-serial fragmentation (diaPASEF) mode, with a scanning range of 349–1229 *m*/*z*, an isolation window of 40 Da, and the collision energy increasing linearly with ion mobility (from 59 eV to 20 eV). A 4D-label-free quantitative proteomics approach was utilized to analyze the protein expression profile of the femoral tissue within the 10–30 kDa molecular weight range. Following a search of standard databases such as UniProt, correlation analysis of replicate samples and evaluation of repeatability were conducted using the R software (version 4.3.1). Differential expression screening was performed using the Benjamini–Hochberg correction, with the criteria of a fold change > 1.2 and an FDR < 0.05. A volcano plot was generated. Additionally, GO functional enrichment (Fisher’s exact test, FDR ≤ 0.05) and KEGG pathway enrichment (Benjamini–Hochberg correction, FDR ≤ 0.05) of the differentially expressed proteins were performed to uncover the functional clustering characteristics and principal metabolic and signal transduction pathways.

### 2.12. Combined Transcriptomic and Proteomic Analysis of Femoral Tissues Treated with the 10–30 kDa Fractions

A correlation analysis was conducted on the transcriptomic and proteomic data of the femoral tissues from rats in the 10–30 kDa fraction treatment group. Initially, molecules with significant differences were identified by applying the screening criteria for DEGs (|log_2_FC| ≥ 0.585, FDR < 0.05) and differentially expressed proteins (DEPs) (|FC| ≥ 1.2, FDR < 0.05). Common gene/protein identifiers from both omics were matched, and a nine-quadrant graph was constructed to illustrate the relationship between mRNA and protein expression levels. Molecules with consistent expression changes in both omics (i.e., co-upregulated and co-downregulated) were selected for further analyses. KEGG pathway enrichment analysis (Fisher’s exact test, FDR ≤ 0.05) was employed to annotate the functions of the co-expressed genes, and significantly enriched signaling pathways and key targets were identified. Through this joint analysis, the core genes regulated by *H. erectus* active peptides at both the transcriptional and post-transcriptional levels and the biological pathways in which they were involved were identified.

### 2.13. Statistical Analysis

All experimental data were presented as mean ± standard deviation (mean ± SD). Each experimental group consisted of at least three independent biological replicates. Normality and homogeneity of variance were evaluated using IBM SPSS Statistics (version 22.0). For data satisfying parametric test assumptions, intergroup comparisons were conducted by one-way analysis of variance (one-way ANOVA), followed by the least significant difference (LSD) post hoc test, and Tukey’s post hoc test was simultaneously performed for result verification. For data that did not meet the assumptions of normality or homogeneity of variance, the Kruskal–Wallis nonparametric test was applied for intergroup comparisons. Statistical significance was defined as * *p* < 0.05, ** *p* < 0.01, *** *p* < 0.001, and **** *p* < 0.0001. All figures were prepared using GraphPad Prism version 10.1. In the bar graphs, error bars denote the standard deviation (SD), and significant differences between groups are indicated with appropriate annotations above the bars.

## 3. Results

### 3.1. Molecular Weight Distribution of Digested Aqueous Extract Products

The dried bodies of *H. erectus* were extracted with water and then freeze-dried, yielding a yellow powder (Supplementary Figure S1a,b). From 1.5 kg of powdered *H. erectus*, 267.5 g of freeze-dried powder was obtained via aqueous extraction and lyophilization, resulting in an overall extraction yield of 17.83%. The simulated gastrointestinal digestion products of HAE exhibited molecular weights of less than 40 kDa. The identifiable bands were concentrated in three regions: a dense band region with low molecular weight (<10 kDa), a diffuse band region with medium molecular weight region (10–20 kDa), and a high molecular weight region (25–40 kDa) (Supplementary Figure S1c). Based on this molecular weight distribution pattern, ultrafiltration membranes with molecular weight cut-offs (MWCOs) of 30 and 10 kDa were used to separate the digest into three fractions: high-molecular-weight (>30 kDa), medium-molecular-weight (10–30 kDa), and low-molecular-weight (<10 kDa). After freeze-drying, the dry weights of these fractions were 155.56, 50.64, and 22.31 g. The yield increased with increasing molecular weight of the fraction (Table 2). All fractions were presented as yellow powders after lyophilization, and the color intensity gradually diminished as the molecular weight decreased (Supplementary Figure S1d).

### 3.2. HPLC Identification and UHPLC-Q-Exactive Orbitrap MS Profiling of Four Fractions

**Figure 1:**
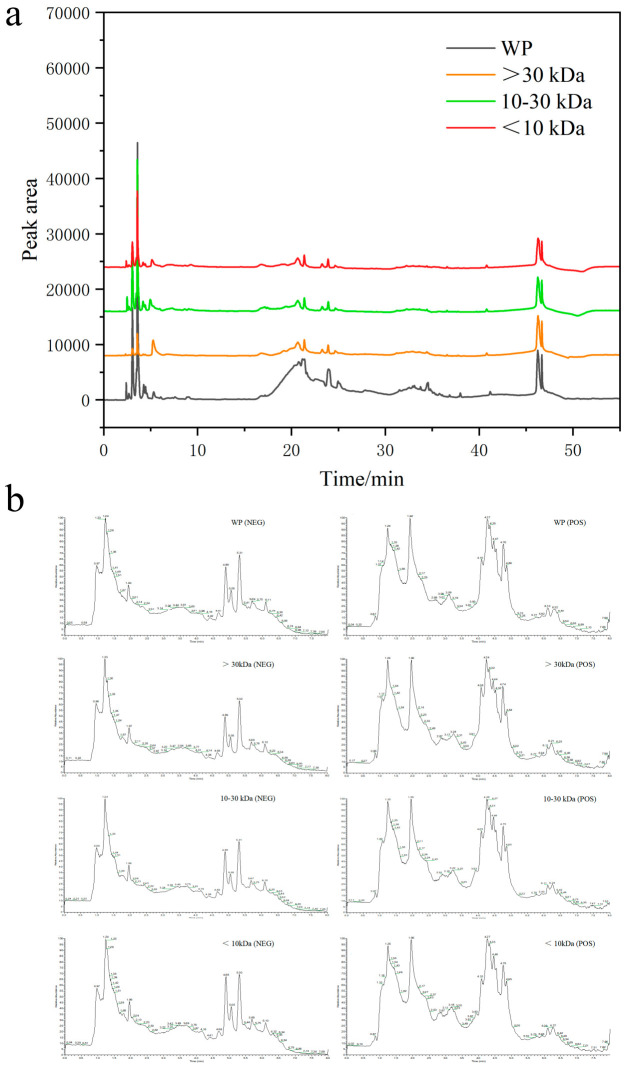
HPLC Identification and UHPLC-Q-Exactive Orbitrap MS Profiling of Four Fractions. Note: (**a**) HPLC chromatograms of the four fractions; (**b**) Total ion chromatogram (TIC) profiles of the four fractions in positive and negative ionization modes.

The total simulated gastrointestinal digest of HAE and its three ultrafiltration fractions were analyzed by high-performance liquid chromatography under optimized conditions. The results showed that all four fractions shared three common characteristic peaks at retention times of 8.2 min, 14.6 min and 22.3 min, indicating the presence of conserved core components across different molecular weight ranges (Figure 1a). Total ion chromatograms (TICs) of the four fractions were generated by UHPLC-Q-Exactive Orbitrap MS in both positive and negative ionization modes (Figure 1b). All fractions showed well-separated ion signals mainly within 1.0–8.0 min, with higher intensities in positive mode. The whole digest (WP) exhibited the most complex profile, while the >30 kDa fraction showed a highly similar TIC pattern to WP, consistent with their dominant hemocyanin subunit composition; hemocyanin subunits are resistant to gastrointestinal proteolysis, with documented tissue repair and immunomodulatory bioactivities. In contrast, the <30 kDa fractions displayed distinct profiles. Notably, the 10–30 kDa fraction had unique high-intensity peaks at 4.0–5.5 min, corresponding to identified bioactive peptides and steroid derivatives. Its peptides contained significantly higher levels of arginine, valine, methionine and hydrophobic amino acid residues than WP (Table S1).

### 3.3. Promoting Effects of Each HAE Fraction on Osteoblast Proliferation and Differentiation

**Figure 2:**
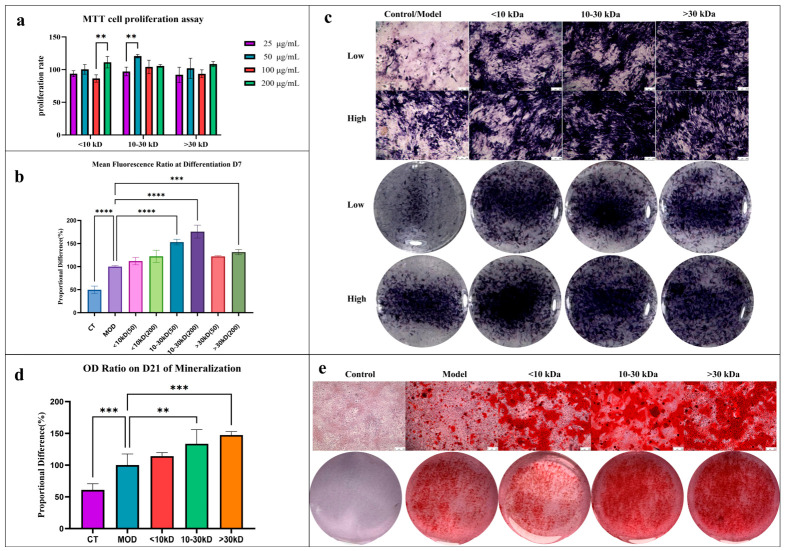
Effects of HAE ultrafiltration fractions on proliferation, differentiation and mineralization of MC3T3-E1 osteoblasts. Note: (**a**) Effects of HAE ultrafiltration fractions on the proliferation of MC3T3-E1 osteoblasts (detected by MTT assay); (**b**) Average optical density value of BCIP/NBT ALP in MC3T3-E1 cells; (**c**) Digital images and micrographs of ALP activity in MC3T3-E1 osteoblasts after 7 days of administration; (**d**) Average optical density value of MC3T3-E1 cells after Alizarin Red staining; (**e**) Digital and micrographs of mineralized nodules in MC3T3-E1 osteoblasts after 21 days of administration. (** *p* < 0.01, *** *p* < 0.001, **** *p* < 0.0001).

MTT assay results indicated that treatment with the 10–30 kDa fraction at 50 μg/mL for 48 h significantly promoted the proliferation of MC3T3-E1 preosteoblasts by 20.7 ± 2.3% compared with the control group (*p* < 0.05; Figure 2a). In contrast, neither the >30 kDa nor the <10 kDa fractions induced significant proliferative effects under identical conditions (Figure 2a). ALP staining and quantitative analysis revealed that after 7 days of treatment with the 10–30 kDa fraction (200 μg/mL), the ALP-positive stained area increased by 75.9 ± 14.0% relative to the osteogenic induction model group (*p* < 0.01; Figure 2b,c), while the ALP staining areas of the <10 kDa and >30 kDa groups showed no significant difference compared with the model group. Alizarin Red staining revealed that the >30 kDa fraction (200 μg/mL) specifically enhanced mineralized nodule formation by 47.3 ± 5.5% compared with the model group (*p* < 0.01), while the 10–30 kDa fraction led to a 33.6 ± 2.3% increase (*p* < 0.05; Figure 2d,e). The area of mineralized nodules in the <10 kDa group showed no significant difference compared with the model group (Figure 2d,e). These results indicated that the >30 kDa fraction exhibited a stronger ability to promote bone matrix calcification.

### 3.4. Effects of HAE on the Body Length, Bone Development and Growth Plate Morphology of Rats

**Figure 3:**
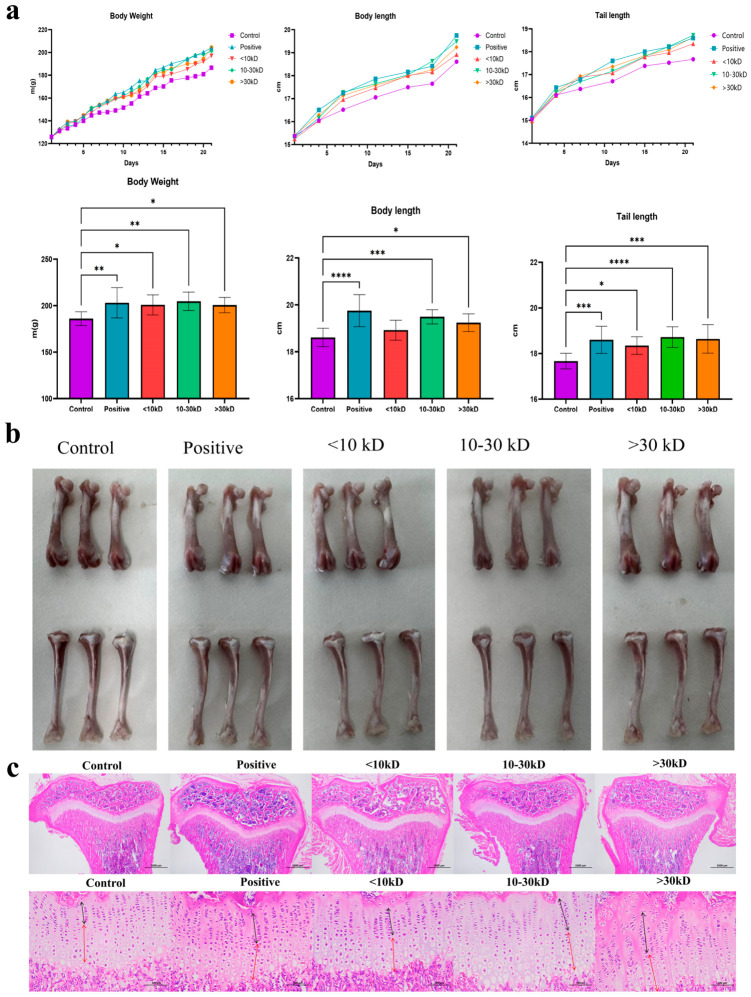
Effects of different molecular weight fractions of HAE on body length, bone development, and growth plate morphology in rats. Note: (**a**) Weight, body length, tail growth trends, and final sampling indicators of rats during the experimental period (10, * *p* < 0.05, ** *p* < 0.01, *** *p* < 0.001, **** *p* < 0.0001). (**b**) Representative images of the tibia and femur in each rat group. (**c**) Morphology of the tibia and growth plate of each rat group under Hematoxylin and Eosin (HE) staining (20× magnification in the upper panel, 200× magnification in the lower panel; the “black arrow” denotes the proliferative zone of the growth plate; the “red arrow” denotes the hypertrophic zone).

Throughout the experimental period, all rats remained in normal physiological condition, with no abnormal feeding behavior or pathological symptoms detected (Figure 3a). On day 9 of treatment, the body weights of all groups, except those treated with the low-molecular-weight (<10 kDa) fraction, were significantly higher than those of the blank control group (*p* < 0.05). At the end of the 21-day intervention, final measurements indicated that, with the exception of the <10 kDa fraction, both body and tail lengths were significantly greater in the treatment groups than in the blank control group (*p* < 0.05). Specifically, the SNL of rats in the 10–30 kDa and >30 kDa groups increased by 5.02 ± 1.42% and 4.78 ± 2.02%, respectively, relative to the blank control group. No statistically significant differences were found between these two groups and the positive control group (6.12 ± 2.78%). Morphological analysis of the bones further corroborated this trend (Figure 3b, Table 3). Compared to the control group, the weights of the tibial diaphysis (302.6 ± 24.5 mg) and Femur diaphysis (370.4 ± 28.6 mg) in the >30 kDa group were significantly increased (*p* < 0.01). Moreover, the length of the tibia (36.18 ± 0.67 mm) was 4.2% greater than that of the control group (*p* < 0.01). In the 10–30 kDa group, both the weights and lengths of the tibia and femur also showed a significant increase (*p* < 0.05). However, there was no significant difference in the <10 kDa group. The observation of growth plate morphology via hematoxylin and eosin (HE) staining and the measurement of zones (Figure 3c, Table 3) revealed that the total heights of the growth plates in the 10–30 kDa group and the >30 kDa group were 356.5 ± 8.4 μm and 373.9 ± 14.3 μm, respectively, which were significantly higher than those of the control group (302.8 ± 5.0 μm, *p* < 0.05). The heights of the proliferative zones (156.5 ± 10.5 μm, 157.6 ± 11.9 μm) in these two groups were significantly higher than those in the control group (114.3 ± 12.9 μm) (*p* < 0.05), and the height of the hypertrophic zone decreased in the >30 kDa group (151.57 ± 10.6 μm).

### 3.5. Effects of HAE on Related Growth Factors in Rats

**Figure 4:**
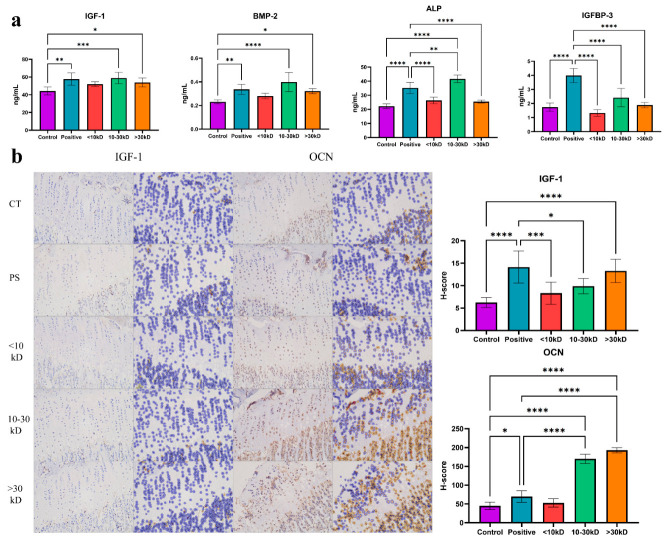
Effects of HAE ultrafiltration fractions on growth factors in Wistar rats. Note: (**a**) Serum IGF-1, BMP-2, ALP concentrations and IGFBP-3 levels; (**b**) IHC staining results of IGF-1 and OCN in growth plate chondrocytes and surrounding trabecular bone regions (*n* = 6, * *p* < 0.05, ** *p* < 0.01, *** *p* < 0.001, **** *p* < 0.0001).

The results of the serum ELISA test (Figure 4a) indicated that the levels of IGF-1, BMP-2, and ALP in the serum of rats in the 10–30 kDa group and >30 kDa group were significantly higher than those in the blank group (*p* < 0.05). Specifically, the ALP activity and BMP-2 levels in the 10–30 kDa group were significantly higher than those in the blank control group (*p* < 0.01), and there was no statistical difference when compared with the positive control group. IGF-1 levels were significantly higher than those in the blank group but were still lower than those in the positive group (*p* < 0.05). Serum IGFBP-3 levels were significantly increased only in the positive control group, indicating a regulatory mechanism distinct from that of other growth factors. IHC staining corroborated these results (Figure 4b). The expression of IGF-1 in growth plate chondrocytes and adjacent trabecular bone regions was markedly upregulated in the 10–30 kDa and >30 kDa groups relative to the blank control, although it was still lower than that in the positive control. Importantly, OCN expression in the 10–30 kDa and >30 kDa groups was significantly higher than that in the blank and positive control groups (*p* < 0.01). Together, these results indicated that active peptide fractions from *H. erectus* may have enhanced longitudinal bone growth and improved bone microstructure via synergistic actions involving systemic hormonal regulation and local factor expression.

### 3.6. The Regulatory Mechanism of 10–30 kDa and >30 kDa HAE on the Development of Rat Femurs Based on RNA Sequencing (RNA-Seq) Analysis

**Figure 5:**
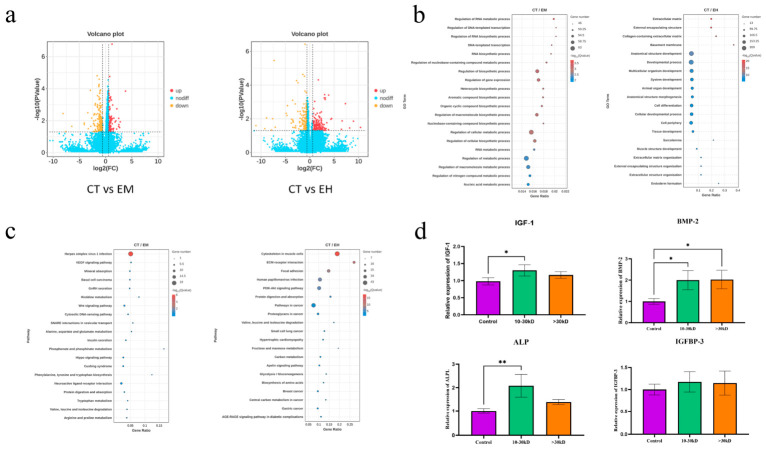
Regulatory mechanism of 10–30 kDa and >30 kDa fractions on rat femur development based on RNA-seq. Note: (**a**) Volcano plot of DEGs; (**b**) Bubble plot of the top 20 secondary functional annotations from GO enrichment analysis of DEGs; (**c**) Bubble plot of the top 20 pathways from KEGG enrichment analysis; (**d**) qRT-PCR validation results of mRNA expression of key bone growth-related genes. CT = control group; EM = 10–30 kDa group, EH = >30 kDa group (*n* = 3, * *p* < 0.05, ** *p* < 0.01).

Based on the in vivo results, we employed RNA-seq to analyze gene expression changes in femoral tissues from the 10–30 kDa group, >30 kDa group and blank control group (CT). Relative to the CT group, the 10–30 kDa group showed 266 DEGs, with 99 upregulated and 167 downregulated. In comparison, the >30 kDa group exhibited 674 DEGs (520 upregulated, 154 downregulated), suggesting that the >30 kDa fraction induced a broader and more pronounced transcriptional response in femoral tissue (Figure 5a). GO enrichment analysis further revealed distinct functional enrichment patterns between the two treatment groups. DEGs in the 10–30 kDa group were mainly associated with RNA metabolic regulation, DNA-templated transcription, and serine-type endopeptidase activity. In contrast, DEGs in the >30 kDa group were significantly enriched in processes related to anatomical structure development, cell differentiation, extracellular matrix organization, and metal ion binding, which are functional categories closely associated with skeletal development and bone remodeling (Figure 5b). KEGG pathway analysis indicated that, in the 10–30 kDa group, DEGs were significantly enriched in pathways such as VEGF signaling, mineral absorption, gonadotropin-releasing hormone (GnRH) secretion, and the Wnt signaling pathway. In contrast, the >30 kDa fraction exhibited significant enrichment in pathways, including extracellular matrix (ECM)–receptor interaction, PI3K-Akt signaling, protein digestion and absorption, and VEGF signaling (Figure 5c). To validate the regulatory effect of HAE, qRT-PCR was conducted to assess the expression of key genes associated with bone growth signaling pathways in Wistar rats. The results indicated that compared with the blank control group, the mRNA expression levels of *IGF-1*, *BMP-2*, and *ALPL* were elevated in the 10–30 kDa group. Moreover, the mRNA expression level of *BMP-2* was significantly upregulated in the >30 kDa group (* *p* < 0.05; ** *p* < 0.01) (Figure 5d).

### 3.7. The Regulatory Mechanism of 10–30 kDa HAE on Femoral Development Based on DIA Quantitative Proteomics

**Figure 6:**
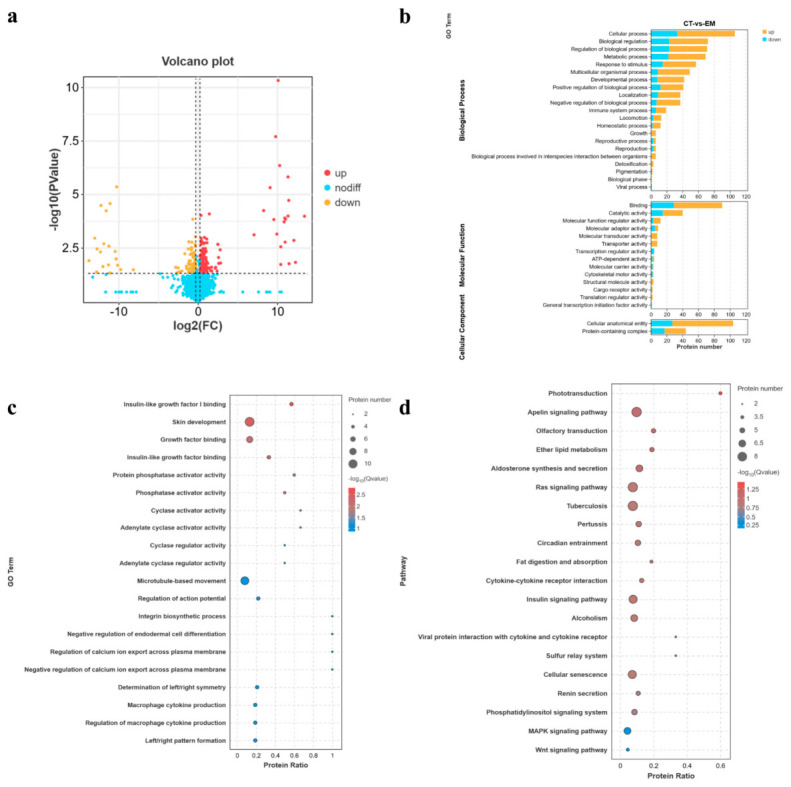
The Regulatory Mechanism of 10–30 kDa HAE on Femoral Development Based on DIA Proteomics. Note: (**a**) Regulation of Differential Proteins. (**b**) First-level functional annotations of the differential proteins in the GO database. (**c**) Bubble chart of the top 20 secondary functional annotations of the differential proteins in the GO enrichment analysis. (**d**) Top 20 pathway analysis of the differential proteins in the KEGG enrichment.

Using DIA quantitative proteomics to analyze femoral tissues from the 10–30 kDa and control groups, we identified a total of 7831 reliable proteins. With a threshold of |FC| ≥ 1.2 and FDR < 0.05, 156 differentially expressed proteins (DEPs) were screened, of which 114 were upregulated and 42 were downregulated (Figure 6a). GO functional annotation revealed that the DEPs were primarily involved in biological processes, such as cellular processes, biological regulation, metabolic processes, and growth. In terms of molecular function, they were significantly enriched in binding, catalysis and structural activities. The cellular fractions were concentrated in the cell anatomical entities and protein complexes (Figure 6b). Secondary function enrichment analysis further demonstrated that the DEPs significantly participated in processes such as insulin-like growth factor I binding (IGF-1 binding), growth factor binding, skin development, and regulation of calcium ion transmembrane output (Figure 6c). KEGG enrichment analysis indicated that the DEPs were significantly enriched in the mitogen-activated protein kinase (MAPK), Apelin, and Wnt signaling pathways (Figure 6d).

### 3.8. Pathways Involving 10–30 kDa Based on Proteome and Transcriptome Combined Analysis

**Figure 7:**
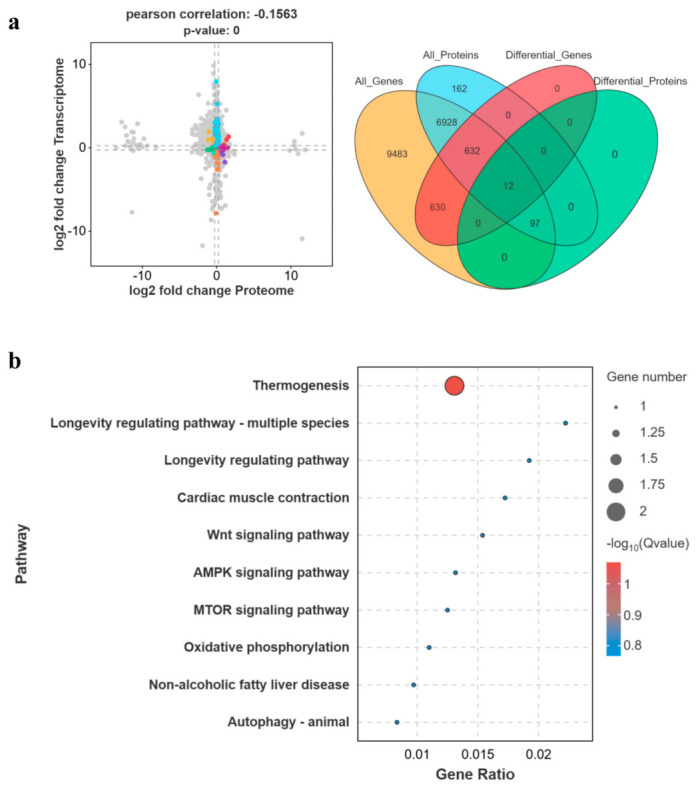
Integrated analysis of the proteome and transcriptome for the pathways of the 10–30 kDa fractions. Note: (**a**) Results of the correlation analysis of co-expressed genes and their expression levels. (**b**) The KEGG enrichment map of co-expressed genes.

To integrate multi-omics data, an association analysis was performed between the transcriptome and proteome of the 10–30 kDa group. The nine-quadrant diagram showed that 12 genes displayed significant differential expression at both the mRNA and protein levels (Figure 7a). Among these genes, the co-upregulated genes were predominantly enriched in Wnt and adenosine monophosphate-activated protein kinase (AMPK) signaling pathways. Further KEGG enrichment analysis identified Frizzled-related protein B (*Frzb*) and AKT1 substrate 1 (*Akt1s1*) as key co-expressed genes that may serve as candidate core targets for the promotion of bone growth by *H. erectus* active peptides (Figure 7b). The results of the combined analysis suggested that *H. erectus* active peptides activated multiple potential core regulatory signaling pathways, such as the IGF-1/PI3K-Akt/Wnt pathway, and jointly regulated gene transcription and protein expression, thereby facilitating endochondral ossification and mineralization of the bone matrix.

## 4. Discussion

Seahorses, traditionally known as “marine ginseng”, have been valued in Chinese medicine for their tonic and therapeutic properties [26]. Classical medical texts and long-standing clinical practices suggest their efficacy in addressing symptoms related to Kidney-Yang deficiency, such as impotence, infertility, enuresis, deficiency-induced dyspnea, soreness and weakness of the lower back and knees, and traumatic injury [27,28]. In coastal regions of China, seahorses have long been incorporated into dietary recipes to promote adolescent growth and development. However, the pharmacodynamic material basis and precise molecular mechanisms underlying this growth-promoting effect remain largely unelucidated in modern pharmacological research. This study systematically investigated the osteogenic and growth-promoting activities of different molecular weight fractions from simulated gastrointestinal digests of *H. erectus* aqueous extract, and further deciphered the underlying regulatory network via multi-omics analysis.

The results of digestion products separation and the LC-MS/MS analysis all indicated that *H. erectus* proteins maintained their structural integrity after simulated digestion. Both WP and >30 kDa fractions were primarily composed of hemocyanin subunits — large copper-containing proteins that are resistant to gastrointestinal proteolysis and have been reported to exhibit diverse biological activities, including immunomodulation and tissue repair [29,30]. Intact hemocyanin macromolecules themselves possess no direct bioactivity; upon oral administration, they are further hydrolyzed in the gastrointestinal tract to generate absorbable bioactive peptides [31,32]. Furthermore, the protease-resistant structural domains contained in the >30 kDa fraction may traverse the intestinal barrier, or bind to intestinal mucosal receptors and modulate the gut microbiota, thereby indirectly influencing the regulation of bone development, which is consistent with previous findings on marine-derived macromolecular proteins [33]. In contrast, the unique peptide profile of the 10–30 kDa fraction, particularly its enrichment in hydrophobic amino acids compared with WP, may underlie its pronounced osteogenic activity, consistent with the effects of fish-derived bioactive peptides [34].

Osteogenic differentiation includes preosteoblast proliferation, early lineage commitment and terminal matrix mineralization, each stage governed by distinct signaling networks. ALP activity serves as a canonical marker of early osteogenic commitment [35], whereas mineralized nodule formation represents terminal osteoblast maturation and completed matrix calcification [36]. In the current study, the finding that the 10–30 kDa fraction enhanced preosteoblast proliferation and ALP activity while the >30 kDa fraction exerted stronger effects on late mineral deposition revealed a stage-specific regulatory pattern driven by their divergent molecular properties. Medium-sized peptides (10–30 kDa) diffuse efficiently through the pericellular space and engage growth factor receptors to activate IGF-1, MAPK and Wnt pathways, which upregulate core osteogenic transcription factors *Runx2* and *Osterix* [37] to drive early differentiation. In contrast, the >30 kDa fraction, dominated by protease-resistant hemocyanin subunits, promoted mineralization mainly via ECM-receptor interaction, PI3K-Akt and VEGF signaling, which remodel the extracellular matrix niche, upregulate osteocalcin and facilitate ordered calcium phosphate deposition [38]. Additionally, the 10–30 kDa fraction exhibited a concentration-dependent functional transition, reflecting sequential receptor engagement: low concentrations preferentially activated high-affinity mitogenic receptors, whereas higher concentrations further engaged lower-affinity receptors linked to differentiation programs [39,40]. These findings provided a critical experimental foundation for determining appropriate dosages in future animal studies and for developing potential functional products.

In animal studies, both the 10–30 kDa and >30 kDa fractions significantly promoted somatic growth and long bone development in juvenile rats, with efficacy magnitude similar to that of subcutaneously administered rhGH. Although bioequivalence cannot be concluded due to different administration routes, these results fully validated the in vivo growth-promoting activity of orally delivered active fractions and corroborated the traditional medicinal use of *H. erectus*. At the molecular level, elevated serum IGF-1 and BMP-2, together with upregulated OCN in the growth plate, constituted a multi-level regulatory network: IGF-1 drives chondrocyte proliferation and osteoblast activation via endocrine, autocrine and paracrine pathways [41,42], while BMP-2 promotes mesenchymal stem cell osteogenic differentiation and matrix mineralization [43,44]. Notably, OCN expression in the active fraction groups was markedly higher than in the rhGH group, suggesting a distinct mechanism from exogenous growth hormone: rhGH mainly stimulates chondrocyte proliferation systemically through the GH-IGF-1 axis, whereas *H. erectus* active fractions can directly enhance late-stage osteoblast function and matrix mineralization locally, forming a dual regulatory mode of systemic endocrine modulation plus local bone anabolism.

Transcriptomic analysis further revealed the transcriptional basis for the functional divergence between the two active fractions, with their pathway enrichment patterns closely matching the stage-specific osteogenic phenotypes observed in vitro and in vivo. The 10–30 kDa fraction was significantly enriched in pathways governing early skeletal development, including Wnt/β-catenin signaling, VEGF signaling and mineral absorption: the Wnt pathway sustains osteoprogenitor proliferation and stem cell renewal to maintain osteogenic reserve [35]; VEGF mediates endothelial migration and vascular invasion, an essential prerequisite for initiating endochondral ossification [45,46]; and enhanced mineral absorption supplied sufficient calcium and phosphate substrates for subsequent matrix calcification. For the >30 kDa fraction, dominant enrichment was seen in ECM-receptor interaction and PI3K-Akt signaling, both core pathways driving late osteoblast maturation and matrix mineralization [38,47,48,49]. Notably, VEGF signaling was enriched in both fractions but presumably exerts stage-specific roles: it supported initial vascular penetration during cartilage-to-bone transition in the 10–30 kDa group, and coupled angiogenesis with terminal bone matrix mineralization in the >30 kDa group. This pathway-level functional division aligned well with the cellular and somatic phenotypes, verifying that *H. erectus* promoted skeletal development through a coordinated, stage-complementary multi-pathway network, in line with previous findings on marine bioactive peptides [40].

The DIA quantitative proteomics analysis of the 10–30 kDa components revealed that the majority of the differentially expressed proteins showed an upward trend. These proteins were significantly enriched in core skeletal development pathways: IGF-1/growth factor binding proteins modulate endogenous IGF-1 bioavailability to boost growth plate chondrocyte proliferation and osteoblast activity [50]; the MAPK pathway transduces extracellular signals via ERK and p38 axes to regulate osteoblast proliferation, differentiation and matrix synthesis [51,52,53]; and Wnt signaling activation at the protein level governs bone cell fate determination and skeletal development [54,55]. This proteomic profile aligned well with the IGF-1 and BMP-2 alterations observed in cellular and animal assays, clarifying the protein-level mechanism underlying the growth-promoting effect.

Twelve differentially expressed genes were identified from the 10–30 kDa components, among which, *Frzb* and *Akt1s1* might be the key regulatory factors for promoting bone formation and growth in *H. erectus*. Functionally, Frzb acts as a spatial regulator; it shapes a precise Wnt ligand gradient across the growth plate by restricting ligand diffusion, which governs the ordered transition of chondrocytes from the proliferative phase to hypertrophic differentiation and ensures the normal progression of endochondral ossification [54,56]. Akt1s1 couples exogenous bioactive peptide signals with bone anabolic processes, precisely regulating protein synthesis, functional maintenance, and matrix mineralization in osteoblasts [57]. The coordinated regulation of these two targets suggested that the active fractions formed a bidirectional “transcription-translation” regulatory network through the cross-talk of Wnt and AMPK-mammalian target of rapamycin (mTOR) pathways, synergistically promoting chondrocyte proliferation, ordered differentiation and terminal bone matrix mineralization to accelerate longitudinal bone growth. This multi-omics collaborative regulatory pattern aligned with previously reported osteogenic mechanisms of marine bioactive peptides [33,58], and provided multi-level molecular insights into the growth-promoting efficacy of *H. erectus*.

Although this study, grounded in traditional medicinal experience, confirmed the growth-promoting effects of *H. erectus*, elucidating the molecular mechanisms underlying its growth-promoting activity still requires a systematic research approach that includes investigating its apparent growth-enhancing effects, identifying its active growth-promoting components, validating the functions of these components, exploring their effect mechanisms, and assessing their in vivo safety profiles. In this study, we isolated components with varying molecular weights from *H. erectus* and demonstrated that the 10–30 kDa and >30 kDa fractions exhibited growth-promoting effects in rats that are similar to those of recombinant human growth hormone. However, these fractions are complex mixtures comprising proteins, peptides, and small-molecule metabolites. Current chemical characterization has only reached the bulk component level, and no single molecule responsible for the observed growth-promoting effects has yet been identified. Future research will focus on isolating specific active components from the 10–30 kDa and >30 kDa fractions, verifying their functional roles, evaluating safety profiles, investigating bioavailability and final active form in vivo, thereby providing a theoretical foundation for the application of *H. erectus* in functional food development.

## 5. Conclusions

This study employed in vitro simulated digestion, ultrafiltration, comprehensive chemical characterization, cell and animal models, transcriptomics, proteomics, and combined analyses to evaluate the growth-promoting effects of digestive products derived from the water extracts of *H. erectus*. The 10–30 kDa and >30 kDa fractions were confirmed as the core active components with stage-specific osteogenic functions. Both fractions effectively promoted body and bone growth in rats by enlarging the growth plate proliferative zone and upregulating serum IGF-1 and BMP-2 levels. Transcriptomic analysis revealed that the >30 kDa fraction induced broader transcriptional changes associated with skeletal development and bone remodeling. In comparison, the 10–30 kDa fraction coordinately regulated bone growth-related genes and proteins. Multi-omics integration further identified *Frzb* and *Akt1s1* as the candidate core targets of the 10–30 kDa fraction, which promoted bone ossification and matrix mineralization by activating the IGF-1/PI3K-Akt/Wnt signaling pathway. These findings reveal the multi-component and multi-potential core pathway mechanism of *H. erectus* and provide a scientific basis for its traditional application and development as a growth-promoting functional food.

## Figures and Tables

**Table 1:** Real-time primer sequences.

Gene Name	Primer Sequences (5′ to 3′)
*IGF1-F*	CTGGTGGACGCTCTTCAGTT
*IGF1-R*	CTTCAGCGGAGCACAGTACA
*BMP-2* *-F*	GTCCCGACGCTTCTTCTTCA
*BMP-2* *-R*	GCTTCCTGCATTTGTTCCCG
*ALPL* *-F*	GGGTGGGTTTCTCTCTTGGG
*ALPL* *-R*	ATGATGGTTGCAGGGTCTGG
*IGFBP-3-F*	GGCCTGACCTACTTGGGAAC
*IGFBP-3-R*	CGCACCGTTATTTGCGACAT
*β-actin F*	CCCTGGCTCCTCAGCACCAT
*β-actin R*	GCCAGGGCAGTAATCTCCTTCT

**Table 2:** Ultrafiltration results of Digested Aqueous Extract Products.

Number of Component	Molecular Weight of Digestive Products (kDa)	Weight (g)	Yield Rate (%)
1	<10	22.31	9.76
2	10–30	50.64	22.16
3	>30	155.56	68.08

**Table 3:** Effects of HAE on the weight and length of the femur and the height of the growth plate zone of the tibia in rats (*n* = 6).

Group	Weight (mg)		Length (mm)		Overall Growth Plate (μm)	Proliferative Zone (μm)	Hypertrophic Zone (μm)
	Tibia	Femur	Tibia	Femur			
Control	259.5 ± 15.3	328.5 ± 27.1	34.73 ± 0.62	30.65 ± 0.77	302.8 ± 5.0	114.3 ± 12.9	168.1 ± 5.6
Positive	303.3 ± 16.1 **	363.4 ± 22.5 **	36.16 ± 0.47 ***	31.88 ± 0.31 **	362.7 ± 9.6 *	152.2 ± 13.2 *	181.7 ± 10.3 *
<10 kDa	273.0 ± 21.0	348.5 ± 17.7	35.23 ± 0.28	31.31 ± 0.48	302.4 ± 12.5	125.9 ± 10.3	157.6 ± 11.9
10–30 kDa	288.8 ± 14.0 *	362.4 ± 7.6 *	35.90 ± 0.24 **	31.61 ± 0.60 **	356.5 ± 8.4 *	156.5 ± 10.5 *	168.87 ± 9.4
>30 kDa	302.6 ± 24.5 **	370.4 ± 28.6 **	36.18 ± 0.67 ***	31.84 ± 0.55 **	373.9 ± 14.3 *	157.6 ± 11.9 *	151.57 ± 10.6

Note: Compared with the control group, * *p* < 0.05, ** *p* < 0.01, *** *p* < 0.001.

## Data Availability

The original contributions presented in this study are included in the article/Supplementary Materials. Further inquiries can be directed to the corresponding authors.

## Supplementary Materials

The following supporting information can be downloaded at: https://www.mdpi.com/article/10.3390/cimb48070678/s1.
