# Supplementary material for: Unraveling the Skeletal Growth-Promoting Mechanism of the Seahorse Hippocampus erectus: From Active Fraction Screening to Signaling Pathway Regulation
Source: Curr Issues Mol Biol. 2026 Jun 30;48(7):678. doi: 10.3390/cimb48070678 (PMC13406864; doi:10.3390/cimb48070678)
Supplement: Supplementary file 1 [file cimb-48-00678-s001.zip › cimb-4375720-supplementary-3/Supplementary Figure S1.pdf]

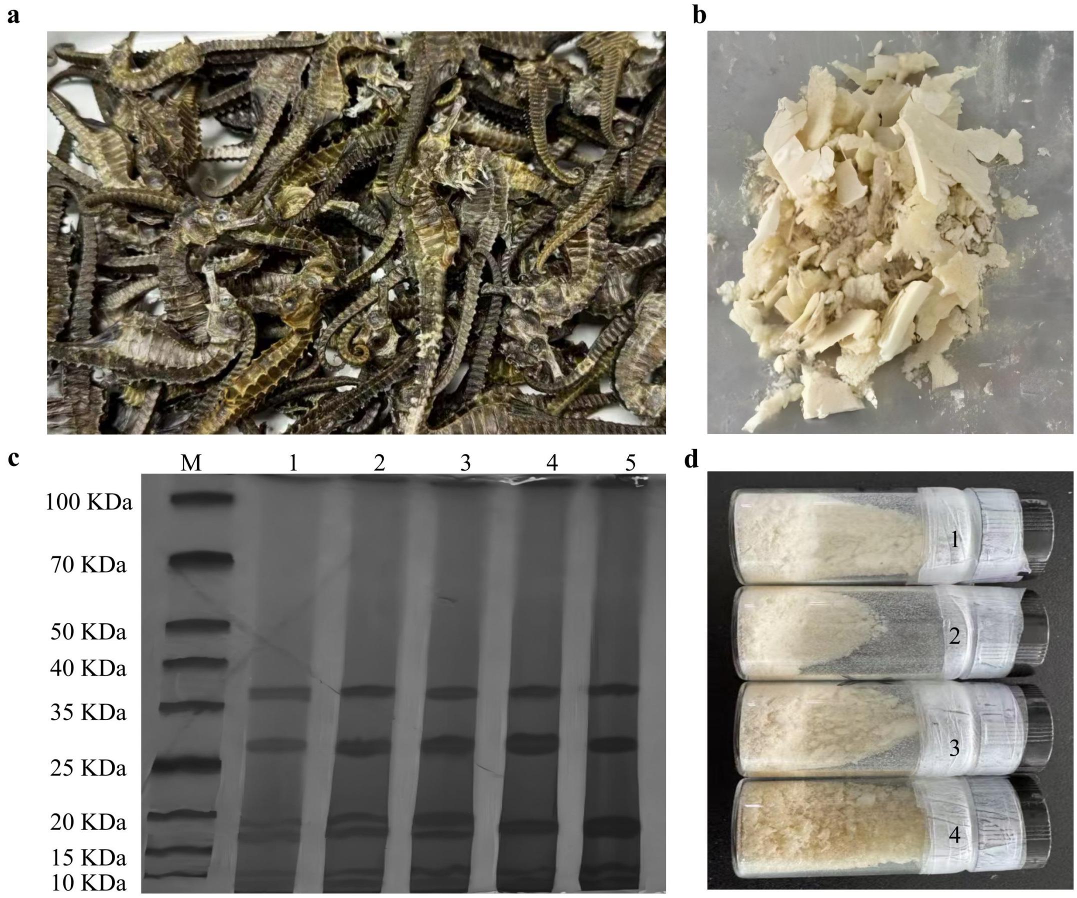

Supplementary Figure S1. Molecular Weight Distribution of Simulated Gastrointestinal Digestion Products and Ultrafiltration Fractionation Results of HAE.

Note: (a) Raw material of *H. erectus*; (b) Freeze-dried powder of aqueous extract; (c) Molecular weight distribution of simulated gastrointestinal digestion products; (d) Freeze-dried powders of ultrafiltration fractions.
